# Disinfection and Care of Broaches

**Published:** 1900-06-15

**Authors:** 


Disinfection and Care of Broaches.—It was the hardest thing I ever had to acquire, the habit of using clean broaches in treating root canals. I keep a dish of pure carbolic acid at hand in which I place my broaches, and after using them I drop them back in the pure carbolic acid. I do not believe in using pure carbolic acid in root canals, but I believe that method of keeping clean broaches is a good one. The carbolic acid is changed every week or ten days and kept nearly pure. It will not rust the broaches, and they will last longer and give more service than if kept in a drier.—*T. B. Hartzell, in Review.*

